# Shielding assessment of a mobile electron accelerator for intraoperative radiotherapy

**DOI:** 10.1120/jacmp.v2i3.2610

**Published:** 2001-09-01

**Authors:** Jodi L. Daves, Michael D. Mills

**Affiliations:** ^1^ Department of Radiation Oncology University of Louisville 529 South Jackson Street Louisville Kentucky 40202

**Keywords:** intraoperative radiotherapy, electrons, shielding, radiation protection

## Abstract

A new approach to intraoperative radiotherapy makes use of a mobile electron linear accelerator delivering therapeutic radiation doses in an operating room suite. This unconventional technology has raised questions concerning protection for personnel and the necessity of shielding the adjoining areas. In this study, the leakage and scatter radiation from the mobile electron accelerator is measured and characterized in a series of spherical projections. An analysis is performed to determine the need for shielding or, alternatively, patient‐based load restrictions in the operating room. This investigation provides a resource to assess shielding and/or patient load restrictions for any facility performing intraoperative radiotherapy with a similar unit. The data presented indicates that the mobile electron accelerator may be operated in an area with little or no shielding under nominal patient load expectations.

PACS number(s): 87.52.–g, 87.53.–j

## INTRODUCTION

**Figure 1:**
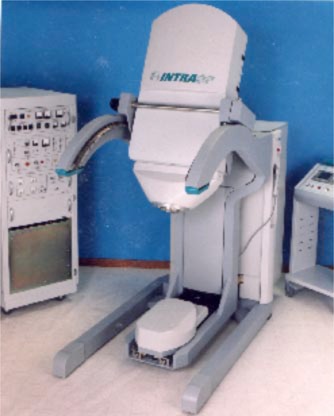
Mobetron.

The Mobetron® (Mobetron is a registered trademark of IntraOp Medical, Inc.) is a Mobile Electron Beam Intraoperative Treatment System designed for electron beam radiotherapy treatments in the operating room.^1^ The attraction of this type of treatment is the ability to deliver a very large uniform dose to a surgically exposed target volume in a single fraction. Doses of 10–25 Gray are delivered while the patient is managed under anesthesia. The electron fields may be collimated and shaped, depending on the clinical presentation. This versatility reduces the dose to the surrounding tissue, as well as to other critical structures. The Mobetron is designed as a mobile unit for Intraoperative Radiotherapy (IORT). Its mobility is possible because of a substantial reduction in weight and size of the accelerator. The size reduction is largely due to its *X*‐band technology and also to the lack of a bending magnet. The highly collimated electron beam, low radiation leakage, and the beam stop are designed to allow the Mobetron to be operated in a room with little or no shielding. The intent of this report is to provide a basis to calculate shielding requirements and/or workload restrictions for the Mobetron. The radiation leakage characteristics and exposure (air kerma) levels are reported in a series of spherical projections. Appropriate use of this data illustrates a standardized method to determine allowable workloads that ensure radiation levels are kept below the regulatory limit while using the Mobetron in any facility. The unit is pictured in Fig. 1.

## MATERIALS AND METHODS

The Mobetron operation results in photon leakage and scatter, as well as electron scatter in the operating room. Due to the limited range of the scattered electrons, the conventional wall material, two sheets of 5/8‐inch drywall, is sufficient to eliminate any radiation hazard from electrons outside the operating room. The photon contamination of the most energetic electron beam, 12 MeV, is around 2 percent. There is only a slight contribution from the Compton interaction within the patient and applicator to the measured photon fluence. Consequently, photon scatter from the patient represents only a small contribution to the overall measured radiation. The primary source of the measured photon fluence is from head leakage. In conventional accelerators the bending magnet is a major source of radiation leakage. The Mobetron uses two *X*‐band (3 cm wavelength 10 GHz frequency) colinear accelerators. This design eliminates the need for a bending magnet, thus effecting a reduction in photon leakage. The Mobetron has two points of potential leakage: the area where the two colinear accelerators meet and the scattering foil.^2^ During measurements, a cylindrical, aluminum applicator with a 10 cm diameter and 32 cm length was attached to a polyethylene phantom with a 13.5 cm diameter and 8.5 cm length. This assembly was used to simulate machine leakage and patient scatter in the treatment configuration. Our measurement surface was a sphere centered on the flattening filter at a radius of 2 meters and bisected by *X Y*, and *Z* planes. The *X* plane, visualized in a top view, contains the measurements that surround the machine at the height of the flattening filter. The *X* plane measurements are applicable to determine the risk of personnel that work on the same floor near the operating room. The *Y* plane is visualized in a side view, while the *Z* plane is visualized in a frontal view of the Mobetron. These measurements help to characterize the radiation risk to floors above and below the unit. The measurement location was chosen to provide optimal geometry for measurement of leakage and scatter from multiple source points along the gantry.

The measurements were taken at the Mobetron production facility (Siemens Medical Systems Oncology Care Systems, Concord, California). During testing, the Mobetron was operated at the calibrated dose rate of 1000 MU/min. for all electron energies, 4, 6, 9, and 12 MeV. The nominal measurements were taken at 2 meters from the scattering foil, in each plane, using a Standard

Imaging Premier 3000 Electrometer (Standard Imaging, Inc., Middleton, WI) and a Capintec Model PM‐30 Ionization Chamber (Capintec, Inc, Ramsey, NJ) with 1 cm buildup cap. Two 5/8‐inch sheets of drywall were placed between the source and the chamber to attenuate scattered electrons. The chamber was secured in position using a multipositional clamp. Readings were corrected for temperature and pressure. The measurements were made every 22.5° in the *X, Y*, and *Z* planes with respect to the gantry. The quality of the leakage and scatter was obtained at two meters and 0 degrees in the *X* plane, which is directly in front of the unit, and at 22.5° in the *Z* plane, which is just lateral to the beam stop. Lead sheets 99.9% pure, with a thickness from 0 to 60 mm, were used to determine the quality of the scatter and leakage.

## RESULTS

For a given energy, the radiation quality two meters in front of the unit was equivalent to the quality just lateral to the beam stop. Beam quality results are presented in Table I.

**Table I:** Mobetron combined leakage and scatter radiation quality by energy.

Quality\Energy	4 MeV	6 MeV	9 MeV	12 MeV
First half value layer, mm lead	10.8	12.1	13.3	13.6
Second half value layer, mm lead	14.8	16	16.2	16.5
Tenth value layer, mm lead	45.1	49.3	50.8	51.9

The first and second half value layers (HVL's) and tenth value layers (TVL's) were calculated using a least squares fit analysis of the data. The least squares fit model overestimates the first HVL; however, this model was chosen to provide a conservative estimate for radiation safety and protection purposes.

**Figure 2:**
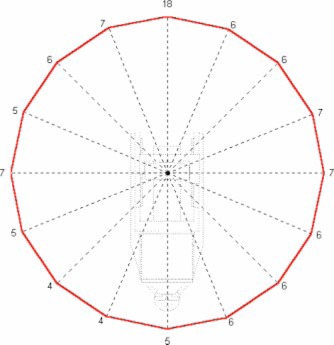
*μ*Gy/1000 monitor units at 2 meters in the *X* plane.

**Figure 3:**
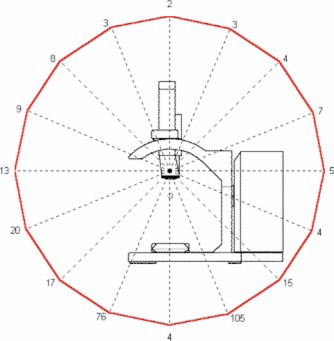
*μ*Gy/1000 monitor units at 2 meters in the *Y* plane.

**Figure 4:**
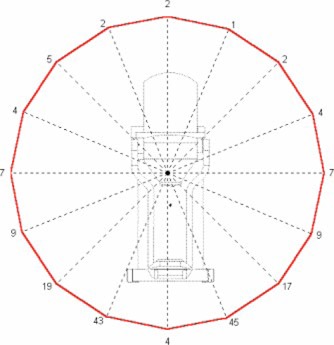
*μ*Gy/1000 monitor units at 2 meters in the *Z* plane.

In this analysis, it was assumed all energies would contribute equally to the workload of the accelerator. All subsequent data presentations average the exposure rate measurements over all energies, thus assuming an equal workload contribution. An average first HVL over all beam energies of 12.4 mm lead was used to calculate the shielding thickness and allowable monitor units. The measurement results for each plane are presented in Figs. 2, 3, and 4. The results are presented in air kerma (*μ*Gy) per 1000 monitor units at two meters from the scattering foil. The measurements are taken every 22.5° in each plane. Given the nominal Mobetron dose rate of $1000\;{\text{MU min}.}^{-1}$, the results also may be presented in $\mu \text{Gy}\;{\text{min}.}^{-1}$ equivalent.

**Figure 5:**
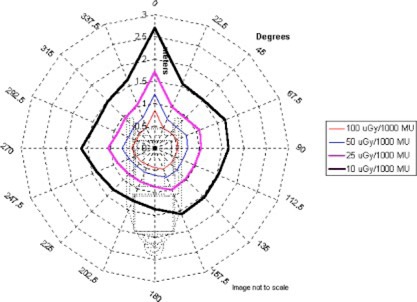
Exposure curves in the *X* plane.

**Figure 6:**
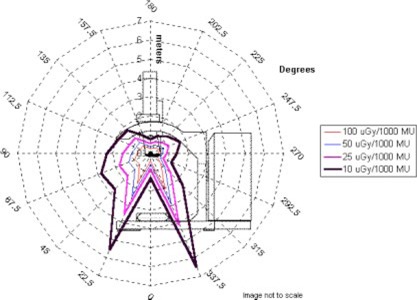
Exposure curves in the *Y* plane.

**Figure 7:**
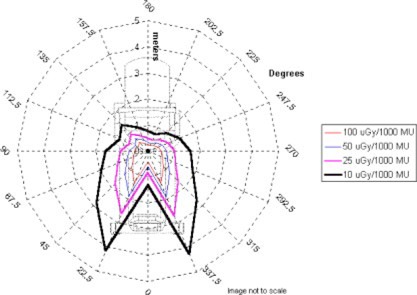
Exposure curves in the *Z* plane.

The $100\;\mu \text{Gy}\;1000\;{\text{MU}}^{-1}$ and 50 $\mu \text{xGy}\;1000{\text{MU}}^{-1}$ exposure curves in each plane are presented in Figs. 5, 6, and 7. The peak exposure occurs at the 0° angle in the *X* plane, which is directly in front of the unit (see Fig. 5). The peak intensity in this plane is most likely due to the leakage from the scattering foil and the colinear accelerator junction. The $100\;\mu \text{Gy}\;1000\;{\text{MU}}^{-1}$ exposure curve occurs at the maximum distance of 0.8 meters and the $50\;\mu \text{Gy}\;1000\;{\text{MU}}^{-1}$ line at 1.2 meters. In the *Y* plane the peak exposures occur at +/−22.5° just lateral to the beam stop (see Fig. 6). The $100\;\mu \text{Gy}1000\;{\text{MU}}^{-1}$ curve occurs at a maximum distance of 2.0 meters and the $50\;\mu \text{Gy}1000\;{\text{MU}}^{-1}$ curve at 2.8 meters.

As expected, the *Z* plane peak exposure occurs again at +/−22.5°, just lateral to the beam stop (see Fig. 7). The $100\;\mu \text{Gy}1000\;{\text{MU}}^{-1}$ line occurs at a maximum distance of 1.3 meters and $50\;\mu \text{Gy}\;1000{\text{MU}}^{-1}$ at 1.9 meters. The peak intensity in these areas for the *Y* and *Z* plane is due to forward scatter, originating in the phantom and applicator, which is not attenuated by the beam stop.

From our measurements we were able to calculate an allowable workload in monitor units per week for all occupied areas surrounding the Mobetron. The calculations are based upon an average air kerma for all energies weighted equally and all treatment positions weighted equally. The allowable monitor unit calculations are derived from the inverse square law and half value thickness for each material. Table II presents workload limits in monitor units per week for the adjoining areas on the same floor. The calculations include workloads for both controlled and noncontrolled areas. Based upon current regulatory limits, the allowable exposure level for noncontrolled areas is considered to be 1 mSv ${\text{year}}^{-1}$, which corresponds to approximately 0.02 mSv ${\text{week}}^{-1}$.^3^ In addition, considering occupancy, in a noncontrolled area no more than 0.02 mSv is allowed during any one hour. The regulatory limits allow controlled areas an exposure 50 mSv ${\text{year}}^{-1}$.^4^ This limit corresponds to an exposure of 1 mSv ${\text{week}}^{-1}$. Controlled areas must be labeled appropriately and access for members of the public should be limited. Occupancy factors were all considered to be in unity for this analysis.

**Table II:** Allowable monitor units per week for walls.

Lead (mm)	Concrete (mm)	Noncontrolled				
		Distance to occupied area (meters)				
		1	2	3	4	5
0	0	600	2400	5 400	9 500	15 000
5	25	800	3100	7 100	13 000	19 600
10	50	1000	4200	9 300	17 000	26 000
15	75	1400	5500	12 000	22 000	34 000
20	100	1800	7200	16 000	29 000	45 000
25	125	2400	9600	22 000	38 000	59 800

1000 MU/min						
Controlled						
Lead (mm)	Concrete (mm)	Distance to occupied area (meters)				
		1	2	3	4	5
0	0	29 700	120 000	270 000	480 000	740 000
5	25	39 000	160 000	350 000	630 000	980 000
10	50	52 000	210 000	470 000	830 000	1 300 000
15	75	69 000	270 000	620 000	1 100 000	1 700 000
20	100	91 000	360 000	810 000	1 400 000	2 300 000
25	125	120 000	480 000	1 100 000	1 900 000	2 980 000
1000 MU/min						

Table III represents workload limits for the areas below the Mobetron and Table IV represents the areas above. All calculations in Tables III and IV were made using an occupancy factor of one. Modifications with respect to occupancy can be made easily using these tables by dividing the allowable workload by the occupancy factor associated for the adjacent area.

**Table III:** Allowable monitor units per week for floor below.

Lead (mm)	Concrete (mm)	Noncontrolled				
		Distance to occupied area (meters)				
		1	2	3	4	5
0	0	70	300	600	1000	1600
5	25	90	400	800	1400	2100
10	50	120	500	1000	1800	2800
15	75	150	600	1400	2400	3700
20	100	200	800	1800	3200	5000
25	125	300	1100	2400	4200	6500

1000 MU/min						
Lead (mm)	Concrete (mm)	Controlled				
		Distance to occupied area (meters)				
1	2	3	4	5
0	0	3 200	13 000	29 000	52 000	81 000
5	25	4 300	17 000	38 000	69 000	110 000
10	50	5 600	23 000	51 000	90 000	140 000
15	75	7 500	30 000	67 000	120 000	190 000
20	100	10 000	40 000	89 000	160 000	250 000
25	125	13 000	52 000	120 000	210 000	330 000
1000 MU/min						

**Table IV:** Allowable monitor units per week for ceiling.

Lead (mm)	Concrete (mm)	Noncontrolled				
		Distance to occupied area (meters)				
		1	2	3	4	5
0	0	2200	8 800	19 800	35 000	55 000
5	25	3000	12 000	26 000	46 000	73 000
10	50	3800	15 000	35 000	61 000	96 000
15	75	5100	20 000	46 000	81 000	130 000
20	100	6700	27 000	60 000	110 000	170 000
25	125	8800	35 000	79 500	140 000	220 000

1000 MU/min						
Lead (mm)	Concrete (mm)	Controlled				
		Distance to occupied area (meters)				
		1	2	3	4	5
0	0	110 000	440 000	990 000	1 800 000	2 700 000
5	25	150 000	580 000	1 300 000	2 300 000	3 600 000
10	50	190 000	770 000	1 700 000	3 100 000	4 800 000
15	75	250 000	1 000 000	2 300 000	4 100 000	6 300 000
20	100	330 000	1 300 000	3 000 000	5 400 000	8 400 000
25	125	440 000	1 800 000	3 970 000	7 100 000	11 000 000
1000 MU/min						

Prior to use at the University of Louisville Hospital, a protection survey was performed for the two operating room suites proposed for Mobetron procedures. The measurements were performed at 22 surrounding locations for each room and readings for all energies were averaged. The protection survey data are presented in Table V for six locations that approximate the 0°, 90°, 180°, and 270° positions in the *X* Plane. The 22.5° and 337.5° positions averaged in the *Y* and *Z* planes correspond to the floor below, and the 157.5°, 180°, and 202.5° positions averaged in the *Y* and *Z* planes correspond to the floor above. The predicted exposure rate values correspond well with measured values from the protection survey.

**Table V:** Mobetron radiation survey for operating rooms 8 and 5.

Degrees	Expected $\left(\text{mR}\;{\text{hr}}^{-1}\right)$	Measured $\left(\text{mR}\;{\text{hr}}^{-1}\right)$ O.R. #8	Measured $\left(\text{mR}\;{\text{hr}}^{-1}\right)$ O.R. #5
0	24	18	18
90	14	12	18
180	7	7	8
270	31	31	35
Floor above	0.3	0.4	0.4
Floor above	11	8	10

## CONCLUSION

The data and methodology demonstrate that for normal room dimensions the walls and the ceiling pose a minor restriction on patient load. The most restrictive workload values occur at the floor below. If the area is occupied and noncontrolled, assuming standard building material and standard distances, the facility may be restricted to only 2000 to 3000 MU per week. However, this conservative restriction can be averted if the area below the Mobetron is deemed a controlled area, or the occupancy of that area is less than one. The protection survey results obtained at the University of Louisville confirm that the data measured at the Mobetron Production Facility may be used to estimate exposure rate values for a Mobetron site location.

Tables II, III, and IV may be used to determine allowable patient workload values for any facility and any building configuration. Assuming standard building materials, this method demonstrates a conservative workload of 3 to 4 patients per week, including warm‐up. Normal patient loads of 3 patients per week, receiving approximately 20 Gy, including warm‐up, fit well within the confines of workload limitations calculated here. The measurements and subsequent calculations clearly demonstrate that the Mobetron can be operated in a room with little or no shielding.

## ACKNOWLEDGMENTS

The authors express their appreciation to Tom Cook, Service Manager, IntraOp Med. Inc., Rich Simon, VP Operations, IntraOp Med. Inc., and Sarah Hughes, Brachytherapy Technologist, Department of Radiation Oncology, University of Louisville, for their assistance. This investigation was supported by a grant from IntraOp Medical, Incorporated.
